# Supplementary material for: The characteristics and treatment patterns of patients with Parkinson’s disease in the United States and United Kingdom: A retrospective cohort study
Source: PLoS One. 2019 Nov 22;14(11):e0225723. doi: 10.1371/journal.pone.0225723 (PMC6874315; doi:10.1371/journal.pone.0225723)
Supplement: Attachment — Submitted filename: Response to Reviewers 16Oct19 v2 clean.docx [file pone.0225723.s001.docx]

**PONE-D-19-24895
The characteristics and treatment patterns of patients with Parkinson’s disease in the United States and United Kingdom: A retrospective cohort study**

Thank you for forwarding the reviewers’ comments. Please find below our responses to each comment.

| **Comment** | **Author response** |
| --- | --- |
| Additional Editor Comments (if provided):  Interesting and quite useful data to be published about medical treatment of PD in two countries with different health care systems.  It would be quite interesting if the author could provide any type of information on two additional treatment that are sure available in both countries, any kind of surgical and Rehabilitation treatments. The time frame is also very interesting, by means of when such treatment became available in each of the countries. | We do agree that it would have been informative to also include information on surgical and rehabilitation treatments for this patient population.  We did not include rehabilitation treatment as part of the study outcomes as this type of care is usually provided outside of the regular hospital visit. Therefore, rehabilitation treatment is usually not captured in the claims database and may also be under-reported in the CPRD database.  We did not include surgical treatments as patients with Parkinson’s disease are recommended for surgical intervention a few years after they have received medical treatment. Although the follow-up period for patients in the CPRD database is long, the average follow-up time for patients in the MarketScan database is 2 years. Therefore, we would not have enough follow-up time to allow the assessment of surgical interventions in this database. Hence, the study outcomes were restricted to evaluating the first-line treatment and outcomes of the first-line treatment, to be able to describe these outcomes in both databases. |
| Comments to the Author  1. Is the manuscript technically sound, and do the data support the conclusions?  The manuscript must describe a technically sound piece of scientific research with data that supports the conclusions. Experiments must have been conducted rigorously, with appropriate controls, replication, and sample sizes. The conclusions must be drawn appropriately based on the data presented.   Reviewer #1: Yes  Reviewer #2: Yes | No changes required based on the reviewer responses |
| 2. Has the statistical analysis been performed appropriately and rigorously?   Reviewer #1: Yes  Reviewer #2: I Don't Know | No changes required based on the reviewer responses |
| 3. Have the authors made all data underlying the findings in their manuscript fully available?  The [PLOS Data policy](https://eur02.safelinks.protection.outlook.com/?url=http%3A%2F%2Fwww.plosone.org%2Fstatic%2Fpolicies.action%23sharing&data=02%7C01%7Clinda.kalilani%40ucb.com%7C331a8a570c02450d5b4008d74b6c2bc8%7C237582ad3eab4d44868806ca9f2e613b%7C0%7C1%7C637060800277396832&sdata=7VAxcskjjcKpBPIcczYwr49FDj2Ox0ZAIZYM78sEb3I%3D&reserved=0) requires authors to make all data underlying the findings described in their manuscript fully available without restriction, with rare exception (please refer to the Data Availability Statement in the manuscript PDF file). The data should be provided as part of the manuscript or its supporting information, or deposited to a public repository. For example, in addition to summary statistics, the data points behind means, medians and variance measures should be available. If there are restrictions on publicly sharing data—e.g. participant privacy or use of data from a third party—those must be specified.  Reviewer #1: Yes  Reviewer #2: Yes | No changes required based on the reviewer responses |
| 4. Is the manuscript presented in an intelligible fashion and written in standard English?  PLOS ONE does not copyedit accepted manuscripts, so the language in submitted articles must be clear, correct, and unambiguous. Any typographical or grammatical errors should be corrected at revision, so please note any specific errors here.  Reviewer #1: Yes  Reviewer #2: Yes | No changes required based on the reviewer responses |
| 5. Review Comments to the Author  Please use the space provided to explain your answers to the questions above. You may also include additional comments for the author, including concerns about dual publication, research ethics, or publication ethics. (Please upload your review as an attachment if it exceeds 20,000 characters)  Reviewer #1: I consider the article relevant. Despite small differences between the data analyzed in both countries, the results were relevant. This article concludes that it is important to build a robust database for population analysis.  Reviewer #2: The manuscript presents good methodology arguments and presents consistent results. There is Consistency and Cohesion in the writing. It is emphasized that new research needs to be done so that it can have increasingly consistent results, especially with other methodologies. | No changes required based on the reviewer responses |
| 6. PLOS authors have the option to publish the peer review history of their article ([what does this mean?](https://eur02.safelinks.protection.outlook.com/?url=https%3A%2F%2Fjournals.plos.org%2Fplosone%2Fs%2Feditorial-and-peer-review-process%23loc-peer-review-history&data=02%7C01%7Clinda.kalilani%40ucb.com%7C331a8a570c02450d5b4008d74b6c2bc8%7C237582ad3eab4d44868806ca9f2e613b%7C0%7C1%7C637060800277406834&sdata=ZPTm%2FVySXi9jVk54nopmFe7LiTrRY0ZZ5QYvy6HTuFI%3D&reserved=0)). If published, this will include your full peer review and any attached files.   If you choose “no”, your identity will remain anonymous but your review may still be made public.   **Do you want your identity to be public for this peer review?** For information about this choice, including consent withdrawal, please see our [Privacy Policy](https://eur02.safelinks.protection.outlook.com/?url=https%3A%2F%2Fwww.plos.org%2Fprivacy-policy&data=02%7C01%7Clinda.kalilani%40ucb.com%7C331a8a570c02450d5b4008d74b6c2bc8%7C237582ad3eab4d44868806ca9f2e613b%7C0%7C1%7C637060800277406834&sdata=9bmCPzWMD7F9%2FVgO9l9%2F8LNGL1gHBYVh4Dmfbnm2pSE%3D&reserved=0).  Reviewer #1: No  Reviewer #2: No | No changes required based on the reviewer responses |
